# Effect of Promoter Methylation on the Expression of Porcine *MUC2* Gene and Resistance to PEDV Infection

**DOI:** 10.3389/fvets.2021.646408

**Published:** 2021-04-29

**Authors:** Yeyi Xiao, Yajing Zhou, Shouyong Sun, Haifei Wang, Shenglong Wu, Wenbin Bao

**Affiliations:** ^1^Key Laboratory for Animal Genetic, Breeding, Reproduction and Molecular Design of Jiangsu Province, College of Animal Science and Technology, Yangzhou University, Yangzhou, China; ^2^Joint International Research Laboratory of Agriculture and Agri-Product Safety, Yangzhou University, Jiangsu Yangzhou, China

**Keywords:** piglet, PEDV, *MUC2*, DNA methylation, intestinal mucosa

## Abstract

Integrity of the intestinal mucosal barrier is closely related to the occurrence of diarrhea. As an important component protein of the intestinal mucosal barrier, Mucin 2 (MUC2) plays a critical role in preventing the invasion of pathogens, toxins, and foreign bodies. In the present study, we preliminary verified the function of the porcine *MUC2* gene in resisting porcine epidemic diarrhea virus (PEDV) infection and investigated the effect of DNA methylation in the promoter region on *MUC2* gene expression. The results showed that after PEDV infection, the intestinal mucosal barrier was damaged. Moreover, *MUC2* expression was significantly higher in PEDV-infected piglets than in healthy piglets (*P* < 0.01). The mRNA expression of *MUC2* was significantly higher in PEDV-infected IPEC-J2 cells than in non-infected IPEC-J2 cells (*P* < 0.05). Methylation of the mC-5 site in the *MUC2* promoter inhibited the binding of Yin Yang 1 (YY1) to the promoter, down regulated the expression of *MUC2* and increased the susceptibility of piglets to PEDV. In conclusion, this study suggests that *MUC2* plays an essential regulatory role in PEDV infection. High *MUC2* expression improves the resistance of pigs to PEDV infection. The binding of YY1 to the *MUC2* promoter is hindered by the methylation of the mC-5 site, which downregulates *MUC2* expression and ultimately affects the resistance of pigs to PEDV infection.

## Introduction

Porcine epidemic diarrhea is a highly infectious intestinal disease caused by porcine epidemic diarrhea virus (PEDV). PEDV invades the intestinal epithelial cells and destroys the integrity of the intestinal mucosal barrier, causing severe diarrhea and ultimately resulting in the death of the infected pigs (1). Therefore, the integrity of the intestinal mucosa is essential to resist PEDV infection.

The intestine acts as the body's largest immune organ in addition to its functions of digestion and absorption, and a complete intestinal mucosal barrier can resist 99% of infections (2). Mucin is a type of high-molecular-weight protein present in secreted and membrane-bound forms. Mucin 2 (MUC2) produced by intestinal goblet cells was the major secreted mucin to be identified. Together with antibacterial peptides, antibodies, defensins, and water, MUC2 forms the intestinal mucus layer, which constitutes a chemical barrier against external pathogens (3, 4). The intestinal mucus consists of two mucin layers, both of which are comprised of a large quantity of MUC2. In healthy conditions, the intestinal mucus exhibits antibacterial activity and functions to protect the epithelial cells, which limits the entry of microorganisms into small intestinal epithelial cells (5, 6). It is reported that decreased *MUC2* gene expression can induce pathogenic bacteria to invade the biological barrier of the intestinal mucosa (7), indicating that MUC2 plays a decisive part in the biological barrier in the intestine. In addition to form a political protective barrier, MUC2 also has direct antibacterial activity against pathogens. It does not directly kill pathogens or inhaled pollutants but captures them by forming a viscous gel and removes them through mucociliary transport (8). In addition, a previous study has reported an increase in the incidence of colon tumors in *MUC2*-knockout mice, which is similar to the inflammatory response in mice with adenomatous polyposis coli gene mutation (9, 10), indicating that the loss of *MUC2* expression is related to the inflammatory response. Many clinical studies have shown that pathogens and inhaled substances can promote mucin production (8). Overall, overexpression of mucin on the surface of epithelial cells is considered a biomarker of potential disease diagnosis under pathological conditions (11).

The protective roles of mucins against pathogenic infections have been observed in previous reports (12–14). However, there are few studies on the regulatory mechanism of the *MUC2* gene expression. DNA methylation is mediated by the DNA methyltransferase family, which does not alter the original DNA sequence. Abnormal DNA methylation can cause a variety of diseases; low methylation leads to abnormal chromatin structure, whereas high methylation may affect gene expression levels (15, 16). DNA methylation modification is one of the main reasons for the decreased expression of many disease resistance genes. In the present study, we analyzed the relationship between *MUC2* gene expression level and PEDV infection. Furthermore, we determined the methylation level of the CpG island in the *MUC2* promoter region and analyzed the effects of the degree of methylation on *MUC2* expression and the key transcription factors in the promoter region. The purpose of this study was to investigate the regulatory effect of *MUC2* gene epigenetic modification on PEDV resistance.

## Materials and Methods

### Ethics Statement

The animal experiment was approved by the Institutional Animal Care and Use Committee (IACUC) of the Yangzhou University Animal Experiments Ethics Committee (permit number: SYXK (Su) IACUC2012-0029).

### Experimental Materials

The experimental pigs were selected from a farm with an outbreak of porcine epidemic diarrhea. We analyzed the pathogens of PEDV, transmissible gastroenteritis virus (TGEV), porcine rotavirus (PoRV), and porcine deltacoronavirus (PDCoV) of 7-day-old piglets with typical clinical symptoms of epidemic diarrhea and selected four 7-day-old piglets infected with PEDV and four healthy piglets feeding in the same conditions. Our previous findings showed that the PEDV detected in pigs is not specific and belongs to the classic strain CV777 (17). The small intestinal tissues were stored in liquid nitrogen for subsequent use.

### Total DNA and RNA Extraction and cDNA Synthesis

Total DNA was extracted from the duodenum, jejunum, and ileum using a DNA extraction kit (Beijing Tiangen Biochemical Co., Ltd., Beijing, China). The concentration and purity of DNA were determined using 2% agarose gel electrophoresis and NanoDrop 1000, respectively. Total RNA was extracted from the intestinal tissues and cells using TRIzol (Invitrogen, USA). Total RNA purity and concentration were assessed using NanoDrop 1000, respectively. RNA samples were stored at −70°C. RNA was reversely transcribed into cDNA using a reverse transcription kit (Vazyme Biotech Co., Ltd., Nanjing, China). The cDNA synthesis reaction mixture (10 μl) contained 5 × PrimeScript Buffer (2 μl), PrimeScript RT Enzyme Mix I (0.5 μl), Oligo dT (0.5 μl), Random hexamers (0.5 μl), RNA (500 ng), and RNase-free H_2_O (up to 10 μl total volume). The thermal conditions used were as follows: 25°C for 10 min, 50°C for 30 min, and 85°C for 5 min; the cDNA samples were stored at 4°C.

### Quantitative Real-Time PCR

RT-qPCR was conducted using the AceQ qPCR SYBR Green Master Mix (Vazyme Biotech Co., Ltd., Nanjing, China). The PCR reaction mixture contained 2 μl cDNA, 0.4 μl of each primer (10 μmol/L), 10 μl 2 × SYBR Premix ExTapTMII, 0.4 μl 50 × ROX Reference Dye II, and RNase-free ddH_2_O (up to 20 μl total volume). The reaction conditions were as follows: 95°C for 5 min, followed by 40 cycles of 95°C 10 s and 60°C 34 s. The melting curve analysis was performed at 95°C for 10 s, 60°C for 60 s, and 95°C for 15 s.

### Pathogen Identification

We rinsed the intestinal tissue with normal saline, randomly cut the tissue, and extracted the total RNA from the tissue. The samples were tested for the presence of PEDV, TGEV, PoRV, and PDCoV using RT-qPCR. Primer information is provided in Supplementary Table 1.

### Hematoxylin-Eosin Staining of the Small Intestinal Tissue

Small intestinal tissues (duodenum, jejunum, and ileum) were fixed with 10% formalin solution, dehydrated with 70, 80, and 90% alcohol, made transparent by treating with xylene solution, embedded with paraffin, sectioned (Leica Rotary Microtome; Leica Biosystems Co., Ltd., Illinois, Buffalo Grove, USA), and stained with hematoxylin-eosin.

### *MUC2* Expression in Tissues and Cells After Porcine Epidemic Diarrhea Virus Infection

IPEC-J2 cells were cultured at 5% CO_2_ and 37°C in 12-well plates with Dulbecco's modified Eagle medium (DMEM; Gibco., Grand Island, New York, USA) containing 10% fetal bovine serum. When the cell density reached 80%, the cells were infected with 0.1 MOI PEDV for 12 and 24 h. Non-infected cells were used as the control group. Four replicates were established per group. *MUC2* expression in the small intestinal tissue of the diarrhea group and control group and infected cells exposed to different infection periods was detected using RT-qPCR. The fluorescent quantitative primers were designed using Primer 5.0 software; *GAPDH* and *ACTB* were used as internal reference genes (Supplementary Table 2).

### RNA Interference of *MUC2* and Detection of Porcine Epidemic Diarrhea Virus *M* Gene Expression

Three siRNA sequences (si-MUC2-1, si-MUC2-2, and si-MUC2-3) and negative control sequence (si-MUC2-NC; Supplementary Table 3) were designed and synthesized by Gene Pharma Co., Ltd. (Shanghai, China). The siRNAs and negative control group (NC) were transfected into IPEC-J2 cells using Lipofectamine 2000, with three replicates per treatment group. Cells were incubated overnight and analyzed for expression of fluorescein-labeled siRNAs after 24 h. RNA interference efficiency was determined using RT-qPCR, and the treatment group with the highest interference efficiency was selected for lipofection; NC and Control were used as control groups. Cells were infected with 0.1 MOI PEDV, and the DMEM complete medium was changed after 2 h. The cell morphology was observed under the fluorescence microscope (MoticAE31 inverted fluorescence microscope; Motic China Group Co., Ltd., Xiamen, China). The mRNA expression level of PEDV M gene was determined using RT-qPCR after 24 h.

### Methylation of *MUC2* Promoter and Its Relationship With mRNA Expression

The promoter region (2,000 bp) of pig *MUC2* was determined using NCBI (https://www.ncbi.nlm.nih.gov/) and Ensembl (http://asia.ensembl.org/index.html) databases. Methprimer software (http://www.urogene.org/cgi-bin/me-thprimer/methprimer.cgi) was used to predict the CpG island in the promoter region of the *MUC2* gene, and primers used in BSP-PCR were designed [Supplementary Table 4, *MUC2* (BSP)]. The Alibaba2 software (http://gene-regulation.com/pub/programs/alibaba2/index.html) was used to predict the transcriptional elements in CpG islands. The genomic DNA was transformed according to the manufacturer instructions of the D5006 bisulfite conversion kit (Beijing Tianmo Technology Development Co., Ltd., Beijing, China). DNA was amplified using the BSP-PCR primers; the PCR reaction mixture (25 μl) included 1 μl of each primer (10 μmol/L), ZYMO Taq Premix (12.5 μl), DNA (2 μl), and ddH_2_O (up to 25 μl total volume). The reaction conditions were as follows: 95°C for 10 min; 38 cycles of 95°C for 30 s, 52°C annealing for 30 s, and 72°C extension for 35 s; and final extension for 10 min. The PCR product was stored at 4°C. The purified PCR product was ligated into the pMD-19T vector (Bao Bioengineering Dalian Co., Ltd., Dalian, China) overnight at 16°C. The ligation product was transformed into Escherichia coli DH-5α competent cells (Beijing Tiangen Biochemical Co., Ltd., Beijing, China) and plated on ampicillin-containing agar plates at 37°C. After 12-16 h, 20 positive monoclonal colonies were picked out per plate and sent to the company (Songon Biotech Co., Ltd., Shanghai, China) for sequencing. The software QUMA (http://quma.cdb.riken.jp/) was used to determine the methylation ratio of each CpG site, and the positive sequencing results were compared with the reference genome.

### Effect of DNA Methylation on the *MUC2* Gene Promoter Activity

We constructed a recombinant plasmid containing the *MUC2* promoter, and primers to amplify the promoter were designed (Supplementary Table 4, *MUC2*-1). The pGL3-basic vector and PCR products were digested using SpeI and NcoI, and the purified products were ligated and transformed into *Escherichia coli* DH-5α competent cells. Monoclonal colonies were picked out for sequencing, and it was verified whether the recombinant plasmid was successfully constructed. The recombinant plasmid pGL3-*MUC2* was methylated with methyltransferase M.SssI, and the product was purified and recovered using the TIANquick Midi purification kit (Beijing Tiangen Biochemical Co., Ltd., Beijing, China). Next, 100 ng M.SssI-methylated plasmid pGL3-*MUC2*, untreated pGL3-*MUC2* plasmid, empty plasmid pGL3-basic, and 2 ng Renilla luciferase reporter plasmid (pRL-TK) were transfected into IPEC-J2 cells cultured in 12-well plates at 37°C for 48 h, and the fluorescence activity was detected using the dual-luciferase reporter gene system.

### Chromatin Immunoprecipitation-PCR

The Pierce Agarose ChIP Kit (Thermo Fisher Scientific, USA) was used for the chromatin immunoprecipitation (ChIP)-PCR assay. Approximately 80 mg of jejunum tissue samples were cut and placed in pre-chilled phosphate-buffered saline; the tissue samples were then fixed with formaldehyde to lyse the tissue and cross-link protein to DNA. The chromatin was enzymatically digested and incubated with specific anti-YY1 antibodies (Proteintech Group, Inc., USA). The protein-DNA complexes were precipitated, eluted, and de-crosslinked; proteins were digested, and enriched DNA was purified and finally precipitated and eluted for PCR amplification. ChIP-PCR was used to detect the enriched precipitated DNA. The positive control using amplified the DNA precipitated by anti-RNA Polymerase II antibody precipitation obtained DNA as a template, the negative control using rabbit Anti-IgG antibody as a template, whereas the input test group amplified *MUC2* gene using unprecipitated DNA as template. The antibodies used in the positive and negative controls were from the Pierce Agarose ChIP Kit. The primers designed for ChIP-PCR are presented in Supplementary Table 4 [*MUC2* (CHIP-PCR)]. The reaction mixture (20 μl) contained 1 μl of each primer (10 μmol/L), 1 μl DNA, 10 μl 2 × Taq Master Mix, and 7 μl ddH_2_O. The reaction procedure was as follows: 95°C for 5 min; 38 cycles of 95°C for 30 s, 60°C annealing for 30 s, and 72°C for 30 s; and 72°C for 10 min. PCR products were detected by 2% agarose gel electrophoresis and compared with the target sequence on the basis of the sequencing results.

### RNA Interference Transcription Factor YY1 and Detection of *MUC2* Gene Expression

Three siRNA sequences and one negative control sequence were designed for the transcription factor Yin Yang 1 (Supplementary Table 3). Primers were designed and synthesized by Gene Pharma Co. Ltd. (Shanghai, China). si-YY1-1, si-YY1-2, si-YY1-3, and si-YY1-NC were transfected into IPEC-J2 cells, with three replicates in each treatment group, and the expression of fluorescein-labeled siRNAs in the cells was assessed after 24 h. The relative expression of the *YY1* gene was detected by RT-qPCR, and the RNA interference efficiency was determined. The treatment group with the highest interference efficiency was selected for further analysis.

### Statistical Analysis

The RT-qPCR results were analyzed using the 2^−ΔΔCt^ method; SPSS 18.0 software was used to analyze the data. The relative quantitative results were analyzed by using independent sample *t*-test for the three replicates of each set; the results are presented as mean ± standard deviation (Mean ± SD). The correlation between promoter methylation and mRNA expression levels was analyzed using Pearson's correlation test. The relative mRNA expression was analyzed by *t*-test using SPSS 18.0 software.

## Results

### Identification of PEDV

In this study, PCR was used to ensure that the piglets in the normal group were not infected with PEDV and the piglets in the diarrhea group were only infected with PEDV. The results showed that the bands in the diarrhea group were consistent with the expected product size (216 bp), while the normal piglet samples showed no obvious bands (Supplementary Figure 1A). In order to eliminate the interference of other pathogens, we employed agarose gel electrophoresis to detect the other common viral diarrhea pathogens (TGEV, PoRV, PDCoV), and the result showed no obvious bands (Supplementary Figure 1B). These results suggested that the piglets in the diarrhea group were only infected with PEDV, whereas the piglets in the healthy group were not infected with PEDV. Therefore, the samples were deemed suitable to use in the subsequent experiments.

### Observation of Small Intestine Tissue and IPEC-J2 Cells After PEDV Infection

**Figure 1:**
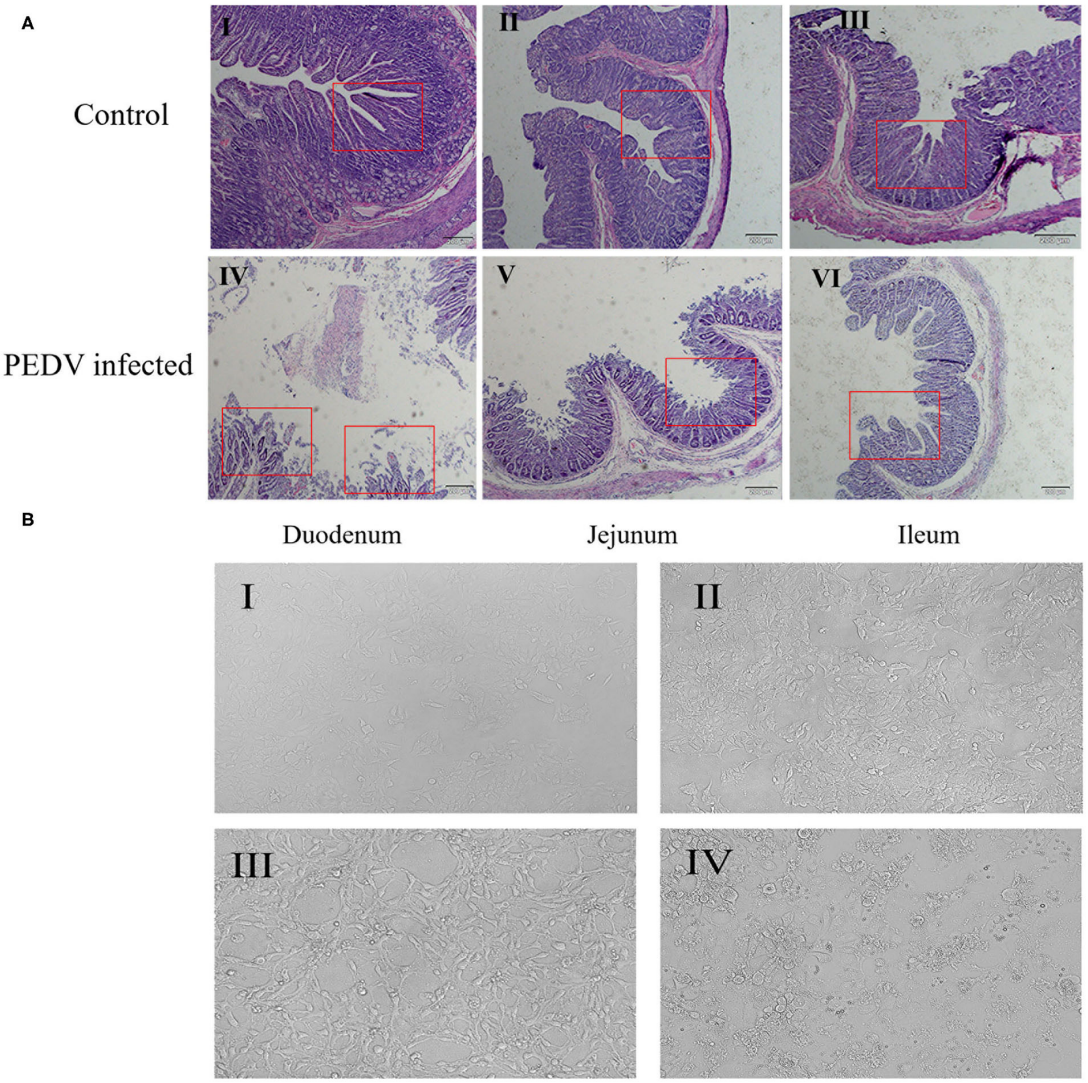
Effects of PEDV infection on intestinal epithelium and IPEC-J2 cells. **(A)** Hematoxylin-eosin staining and microscopic examination of small intestinal tissues (10×). The control group represents the Duodenum (I), Jejunum (II), Ileum (III) of normal piglets, and the PEDV infected group represents the Duodenum (IV), Jejunum (V), Ileum (VI) of diarrheic piglets. The red square marks the changes of intestinal villi. **(B)** Cell morphology of PEDV-infected IPEC-J2 cells at different time periods observed under a light microscope (100×). (I) 0 h, (II) 12 h, (III) 24 h, (IV) 48 h.

PEDV can cause damage to the intestinal barrier, in the present study, we want to verify whether PEDV can also damage the intestinal barrier in pigs. So, we observed the intestinal damage caused by PEDV infection under microscope. As shown in Figure 1A, the intestinal villi of the diarrhea group piglets were structurally damaged: the villous density and height were decreased, the villi become sparse, the lamina propria was exposed, and the intestinal glands were atrophic. In contrast, the intestinal mucosal barrier of healthy piglet was intact, and the intestinal mucosal epithelial cells were well-organized, clear in outline, and regular in arrangement.

At the same time, we observed the damage caused by PEDV to IPEC-J2 cells using optical microscopy. The result showed that, after 24 h of PEDV infection, cell morphology began to change, and numbers of atrophied cell increased (Figure 1B-III), whereas after 48 h of PEDV infection, cell lesions occurred, normal cell morphology was completely lost, and extensive cell death occurred (Figure 1B-IV). These results showed that PEDV could cause damage to IPEC-J2 cells and small intestine tissues in piglets.

### *MUC2* Is Up-Regulated in PEDV-Infected Tissues and Cells

**Figure 2:**
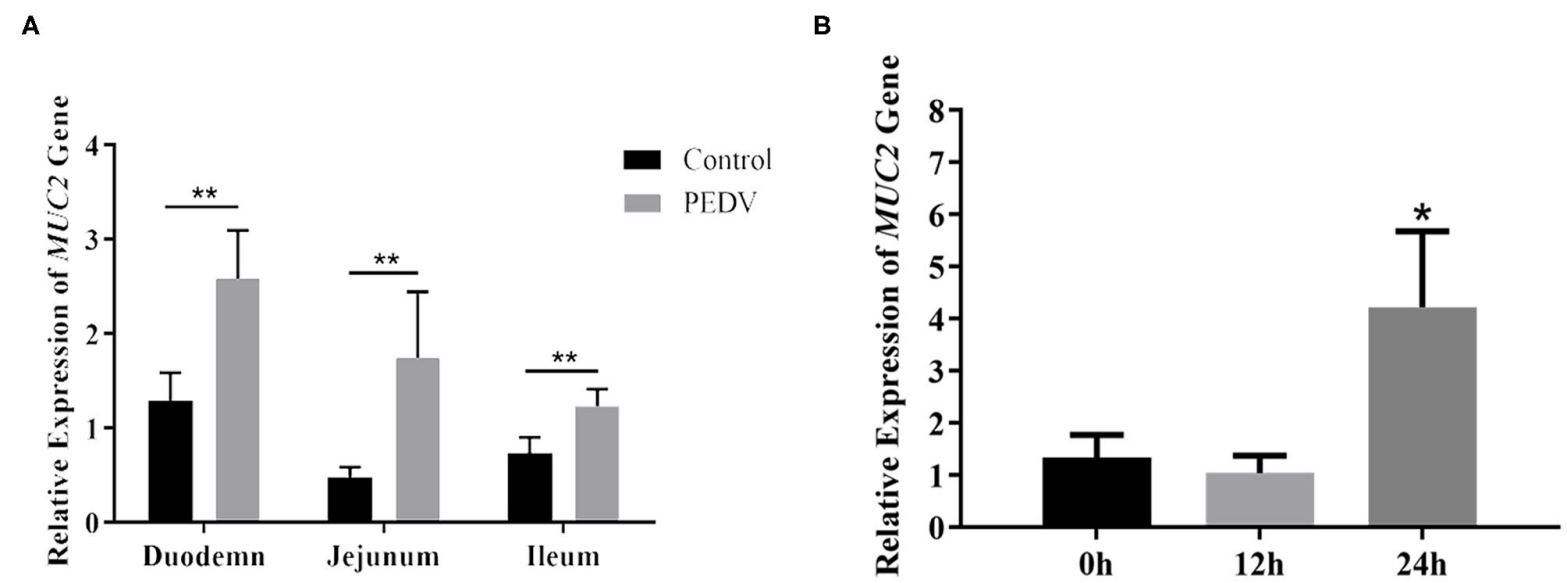
Changes of *MUC2* expression after PEDV infection. **(A)** *MUC2* expression in the small intestine of the diarrhea group piglets and healthy piglets. **(B)** *MUC2* expression in PEDV-infected IPEC-J2 cells at different time periods. **P < 0.01,*P < 0.05.

MUC2 protects the gut from pathogenic microorganisms, so does MUC2 play a role in PEDV infestation? To investigate that, the mRNA level of *MUC2* was detected at the cellular and organizational levels after PEDV infection. The results showed that the *MUC2* expression level in the small intestine (duodenum, jejunum, and ileum) of piglets from the diarrhea group was significantly higher than that from the healthy group (*P* < 0.01; Figure 2A). In addition, the results of PEDV infection of IPEC-J2 cells at different time periods showed that *MUC2* expression was significantly up-regulated at 24 h (*P* < 0.05; Figure 2B). Overall, these results suggested that *MUC2* may play a significant role in PEDV infection.

### *MUC2* Knockdown Contributes to PEDV Replication

**Figure 3:**
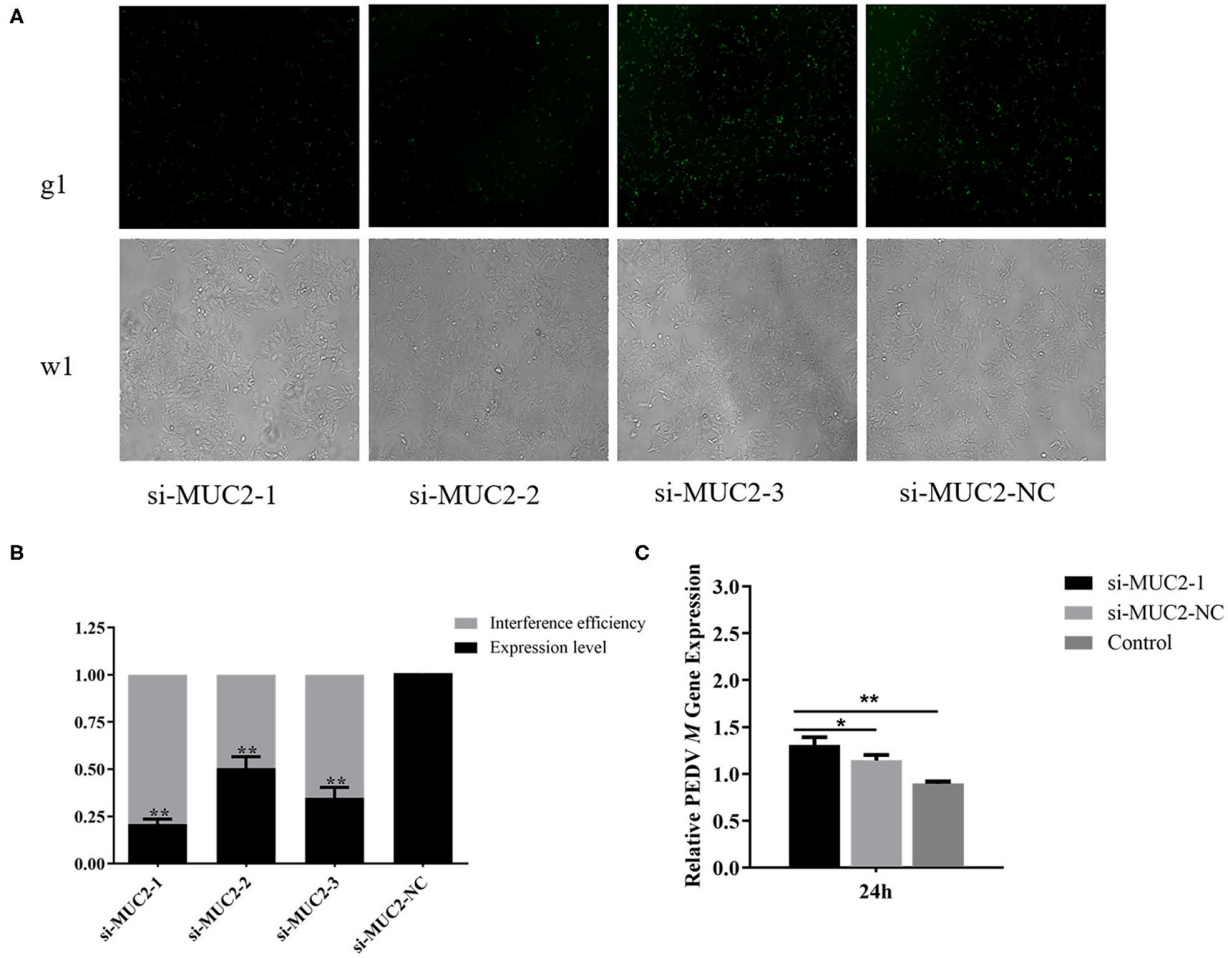
Effects of *MUC2* gene knockdown on PEDV replication. **(A)** The expression of fluorescein markers in IPEC-J2 cells under fluorescence microscope (g1) (100×); the lower panels (w1) are the corresponding cells in the absence of fluorescence exposure, which shows the overall density of cells. **(B)** The mRNA expression of *MUC2* and analysis of interference efficiency. **(C)** Detection of PEDV *M* gene expression after interference with *MUC2* expression. **P < 0.01, *P < 0.05.

To further investigate the role of *MUC2* in PEDV infection, we used RNA interference to down regulate *MUC2* expression. The fluorescein markers, used to monitor siRNA transfection, were highly expressed after 24 h, indicating that siRNAs had been successfully transfected (Figure 3A). The treatment group with the highest interference efficiency (si-MUC2-1) was selected for the subsequent experiments (Figure 3B). Then we detected the PEDV *M* gene to investigated the effect of *MUC2* down regulation on PEDV replication. The result showed that after 24 h of PEDV infection, *M* gene expression in the *MUC2*-RNAi group was significantly higher than that in the *MUC2*-NC group (*P* < 0.05), and significantly higher than that in the Control group (*P* < 0.01; Figure 3C). In general, these results showed that down regulation of *MUC2* expression may contribute to PEDV replication.

### Analysis of DNA Methylation in the Promoter Region of the *MUC2* Gene

To investigate whether DNA methylation is regulating the expression of *MUC2*, we predicted the CpG island in the *MUC2* promoter and the results showed that there was a CpG island ~208 bp in size (−324 to −116 bp; Supplementary Figure 2A). The CpG island was amplified by PCR, and the PCR product was detected by 1% agarose gel electrophoresis. The result showed a single bright band, and the size was as expected (Supplementary Figure 2B).

**Figure 4:**
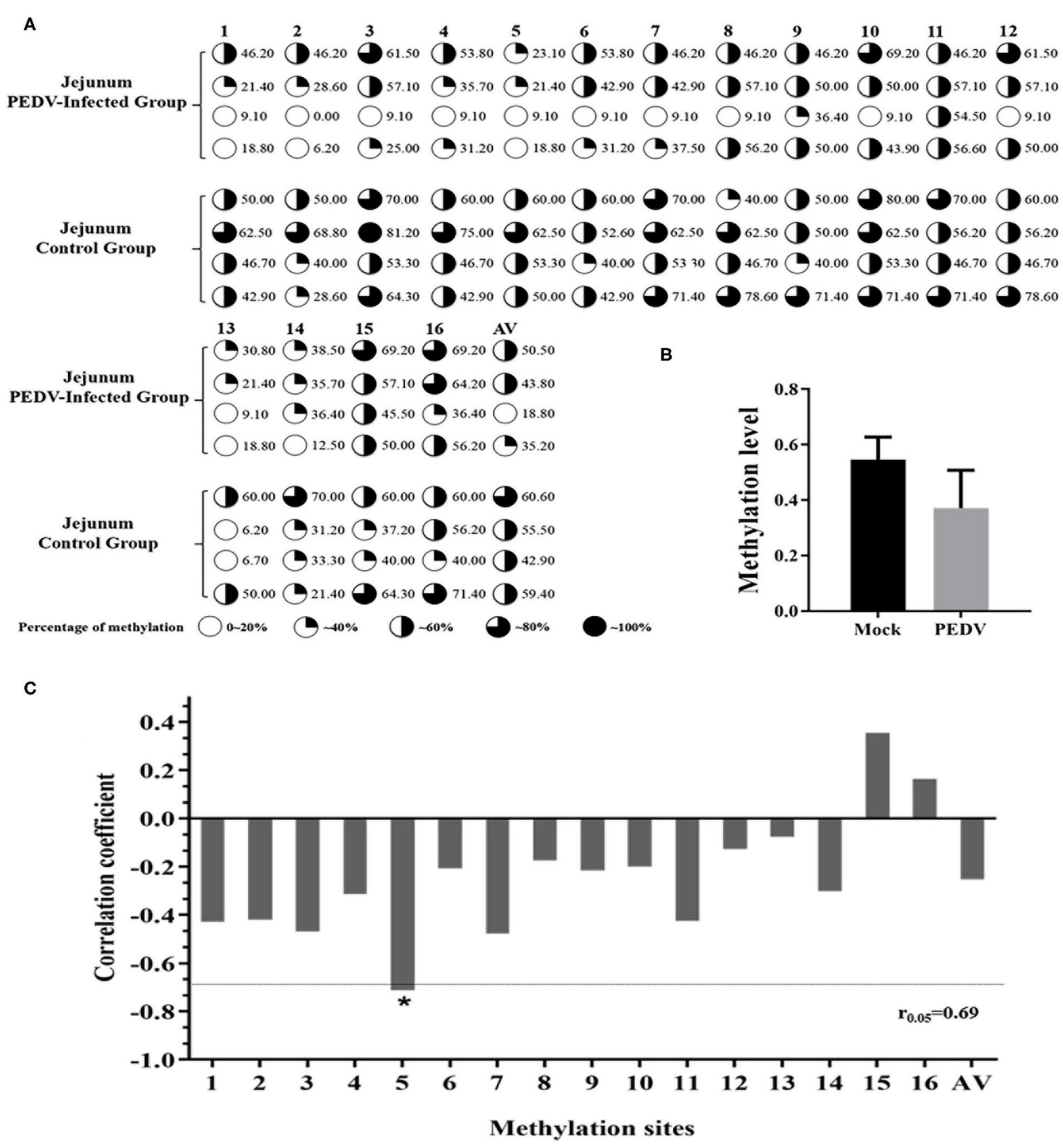
Correlation between the degree of methylation of the CpG island in *MUC2* promoter region and mRNA expression. **(A)** The methylation level of each site of the CpG island of the *MUC2* gene promoter. **(B)** Degree of methylation between the two groups. **(C)** Correlation between the degree of methylation of the CpG island in *MUC2* gene promoter region and mRNA expression. *P < 0.05.

In order to further explore the effect of DNA methylation on *MUC2* expression. We analyzed the correlation between CpG island methylation in *MUC2* promoter and mRNA expression. The results of the sequencing showed that there were 16 CG sites in the CpG island of the *MUC2* promoter region, all of which showed different degrees of methylation (Figure 4A); the methylation level in the diarrhea group was lower than that in the healthy group (*P* > 0.05; Figure 4B). The methylation levels of most sites were negatively correlated with mRNA expression, except mC-15 and mC-16; among these sites, the methylation level of mC-5 was significantly correlated with mRNA expression (*P* < 0.05; Figure 4C).

**Figure 5:**
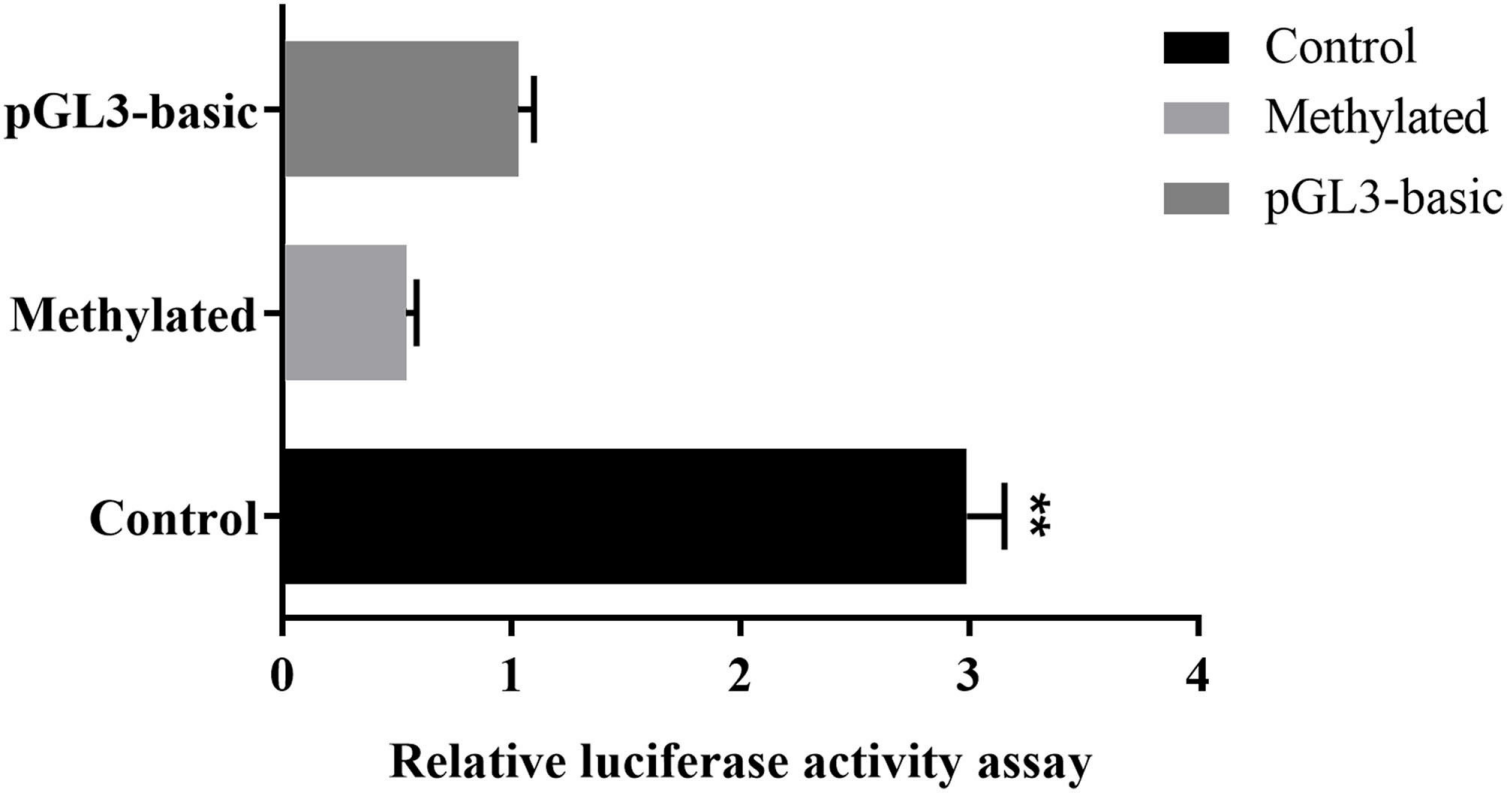
Effects of DNA methylation on the *MUC2* gene promoter activity. pGL3-basic: empty plasmid; Methylated: pGL3-*MUC2* recombinant plasmid were treated with M.SssI; Control: pGL3-*MUC2* recombinant plasmid was not methylated. Both Methylated group and Control group contained the promoter sequence analyzed in this study. **P < 0.01.

To further prove that DNA methylation can affect *MUC2* expression. We detected the effect of promoter methylation of *MUC2* gene on promoter activity using Dual-luciferase reporter assay. The results showed that the methylation of the CpG island of the *MUC2* promoter significantly reduced the luciferase activity (*P* < 0.01; Figure 5), suggesting that DNA methylation inhibited the transcriptional activity of *MUC2*.

### Transcription Factor-Binding Sites Prediction and Confirmation

We next predicted transcription factors in CpG island of *MUC2* promoter region to investigate how the mC-5 site inhibits *MUC2* expression. The result showed that the CpG island of *MUC2* promoter has 14 transcription factors. A Pearson correlation analysis suggested that the methylation level of the mC-5 site in the binding domain of the transcription factor YY1 was significantly negatively correlated with gene expression (*P* < 0.05; Supplementary Figure 3).

**Figure 6:**
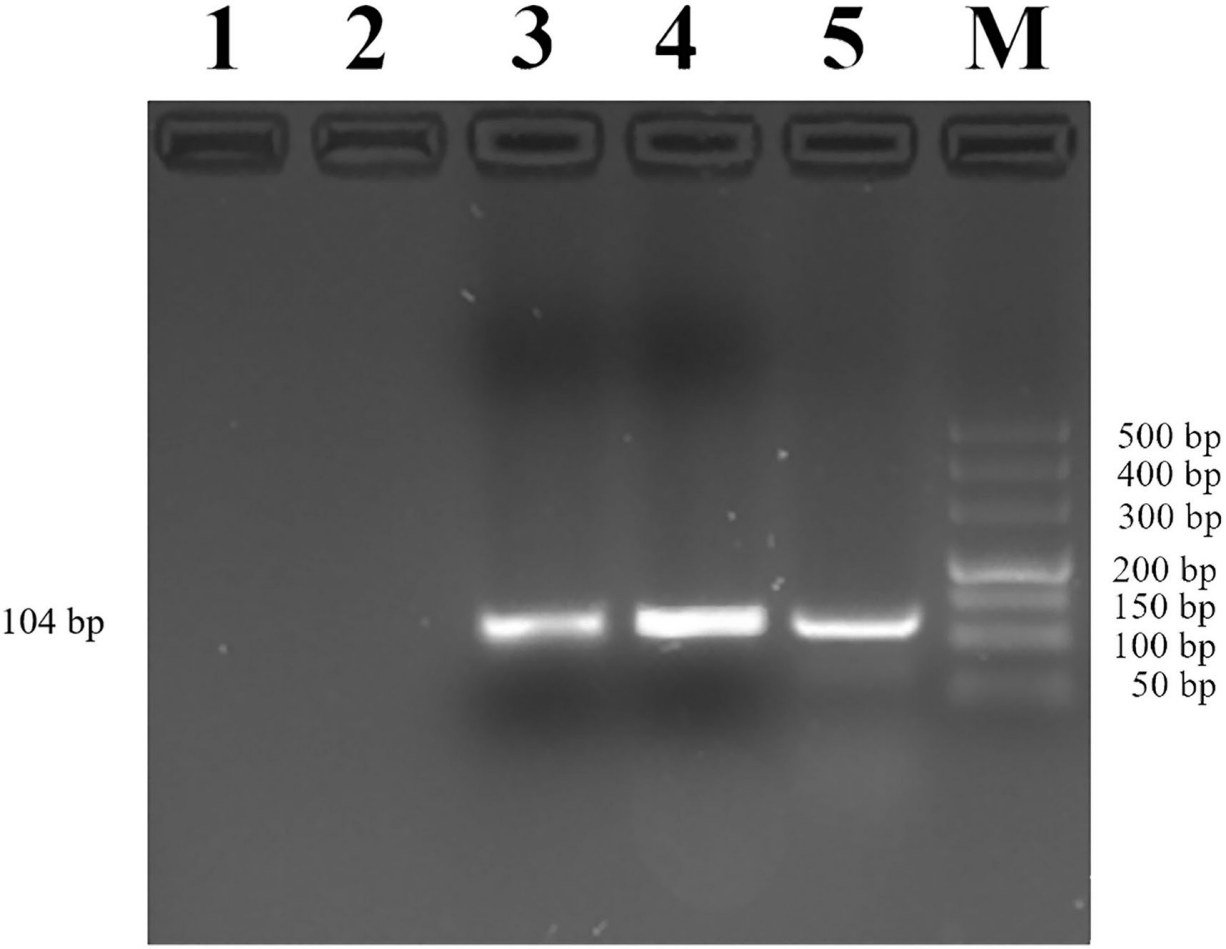
Identification of YY1 binding to the *MUC2* gene promoter. Lane 1: PCR amplification products with RNase-free water (Blank control without DNA template). Lane 2: Rabbit IgG antibody was added before PCR amplification (negative control). Lane 3: Anti-YY1 antibody was added before PCR amplification. Lane 4: PCR amplification product with input. Lane 5: Anti-RNA Polymerase II antibody was added before PCR amplification (positive control). Lane M: DL500 DNA marker.

Then, we used CHIP-PCR to investigate whether transcription factor YY1 binds to the promoter region of *MUC2*. The result showed a single bright band (104 bp), which was consistent with the target fragment in size and with the sequencing results (Figure 6). The results suggested that transcription factor YY1 specifically binds to the mC-5 site of *MUC2* gene promoter region.

### YY1 Knockdown Down-Regulates *MUC2* Expression

**Figure 7:**
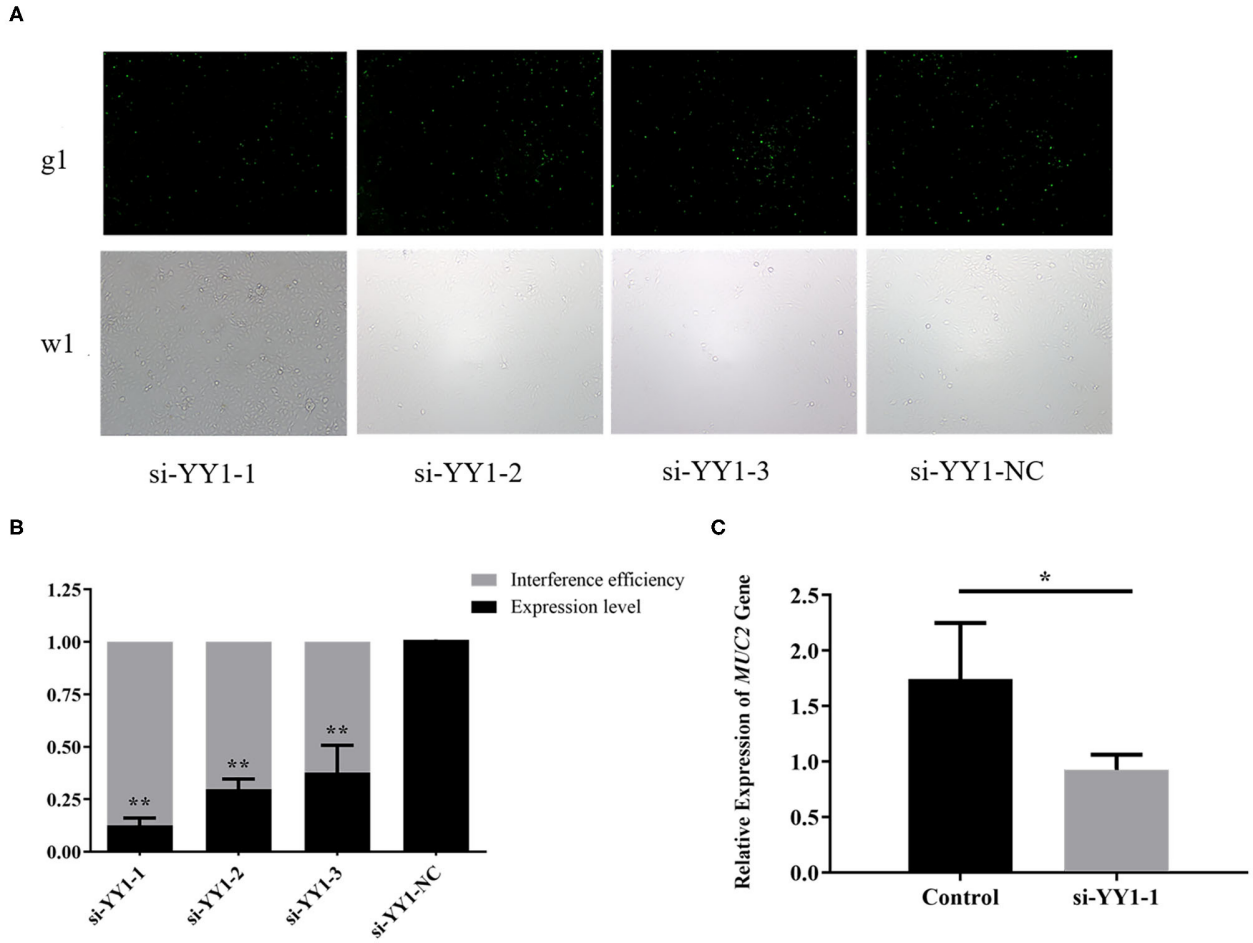
Effects of *YY1* gene knockdown on *MUC2* expression. **(A)** The expression of fluorescein markers in IPEC-J2 cells under fluorescence microscope (g1) (100×); the lower panels (w1) are the corresponding cells in the absence of fluorescence exposure, which shows the overall density of cells. **(B)** The mRNA expression of *YY1* and analysis of interference efficiency. **(C)** The mRNA expression of *MUC2* gene after RNA interference. **P < 0.01, *P < 0.05.

To explore the effect of transcription factor YY1 on *MUC2* gene expression, we constructed the RNA interference vector of *YY1* and transfected it into IPEC-J2 cells. The fluorescein markers were detected to be highly expressed after 24 h, indicating that siRNAs were successfully transfected (Figure 7A). The treatment group with the highest interference efficiency (si-YY1-1) was used to interfere *MUC2* expression. (Figure 7B) RT-qPCR results showed that the expression level of *MUC2* gene decreased significantly (*P* < 0.05; Figure 7C) suggesting that interfering transcription factor YY1 can down regulate *MUC2* gene expression.

## Discussion

The mortality of PEDV infection in piglets within 7 days or even a few hours after birth is 100% (18, 19), which leads to severe economic losses to the pig industry. The small intestine plays a significant role in absorbing nutrients and blocking various antigens entering through the villi and crypts of the intestinal epithelium. PEDV infection can lead to acute and massive necrosis of epithelial cells and severe atrophy of intestinal villi (20–22). Previous studies have revealed that the small intestine of PEDV-infected piglets appears thin and transparent (23), intestinal cells are shed, and enteritis occurs (24). These results are consistent with the observations of intestinal sections in the present study, indicating that PEDV-infected piglets have lesions in the small intestine. Additionally, PEDV causes structural damage to the small intestinal villi and decreases villous height, damaging the intestinal mucosal barrier. Furthermore, PEDV can affect the immune, digestive and absorptive functions of the intestine and eventually cause diarrhea. The results further indicate that the pathogenicity of PEDV depends on the integrity of intestinal mucosal barrier in piglets to a certain extent.

MUC2 is an essential structural component of the intestinal mucosal barrier. An intact mucosal barrier can prevent pathogenic microorganisms in the intestinal cavity from contacting the epithelium. It plays an anti-infective role and regulates the balance between intestinal immunity and external stimuli (25, 26). In the present study, the *MUC2* gene expression in the small intestinal tissue of PEDV-infected piglets was noted to be significantly higher than that in the intestine of healthy piglets, indicating that PEDV infection was associated with high *MUC2* expression. In addition, 24 h after the PEDV infection of IPEC-J2 cells, the *MUC2* expression was significantly up-regulated. Moreover, at this time point, the cell morphology began to change. These results indicated that the up-regulated expression of *MUC2* is advantageous in repairing the damaged intestinal mucosal barrier. And the MUC2 protein in the intestinal lumen can remove the pathogens to protect the intestinal epithelium from pathogen invasion. Furthermore, it was observed that the expression level of PEDV *M* gene in IPEC-J2 cells significantly increased after *MUC2* knockdown. Previous studies have shown that after *MUC2* knockdown, intestinal MUC2 secretion is reduced, and the mucus layer becomes thin and discontinuous (27). Our results suggested that the lack of MUC2 protein leads to impaired function of the intestinal mucosal barrier and induces PEDV to invade the mucosal lamina propria, resulting in a surge of PEDV replication. Overall, our data indicated that high *MUC2* expression is beneficial to resist PEDV infection.

In the present study, we found that there were 16 CG sites in the promoter region of *MUC2* and all of them had different degrees of methylation. Correlation analysis of *MUC2* mRNA expression showed that the methylation of most sites was negatively correlated with mRNA expression. Among these sites, the methylation of mC-5 site was significantly negatively correlated with *MUC2* mRNA expression. The result of dual-luciferase reporter assay indicated that DNA methylation inhibits the transcription of *MUC2*. These results suggested that the mC-5 site may be an important methylation site that regulates *MUC2* gene transcription.

DNA methylation is associated with gene transcription silencing (28–30). Blocking the binding of transcription factors and promoters is one of the mechanisms that inhibit gene expression. By inhibiting the binding of methylation-sensitive transcription factors to DNA or hindering the binding of methylation-insensitive transcription factors through proteins, the transcription and expression of the gene can be regulated by DNA methylation (31). In the present study, we identified 14 potential transcription factor-binding domains in the CpG island region of the *MUC2* promoter, of which the mC-5 site was located in the transcription factor YY1-binding domain. YY1 is a zinc finger transcription factor widely expressed in various tissues and participates in various biological processes such as embryonic development and differentiation, tumor metastasis, and cell proliferation (32). As a transcription factor, YY1 can act as an activation factor to activate gene expression or as an inhibitor to suppress gene transcription (33, 34). In the present study, the result of ChIP-PCR confirmed that the transcription factor YY1 specifically binds to the *MUC2* gene promoter. Moreover, the expression level of *MUC2* decreased with the down-regulation of *YY1* expression. This indicated that methylation of the mC-5 site hinders the binding of the transcription factor YY1 to the promoter target sequence, thereby inhibiting the expression of *MUC2*.

In this study, we verified that high expression of *MUC2* contributes to the resistance of piglets to PEDV infection. Meanwhile, DNA methylation was found to be associated with MUC2 expression. It is worth noting that methylation of mC-5 site which is located in the binding area of YY1 was significantly negatively correlated with *MUC2* expression. We speculated that DNA methylation reduces the transcriptional activity in the promoter region of *MUC2*, and the mC-5 methylation of the *MUC2* promoter inhibits its binding with YY1, which further inhibits *MUC2* expression and ultimately regulates the ability of piglets to resist PEDV infection. This study verified the role of MUC2 in PEDV infection, and revealed the regulatory mechanism of *MUC2*, which lay the foundation for deeply exploring the regulatory mechanism of *MUC2*, and further functional and mechanism studies on this gene in pigs will be our further endeavor.

## Data Availability Statement

The original contributions presented in the study are included in the article/Supplementary Materials, further inquiries can be directed to the corresponding author/s.

## Ethics Statement

The animal experiment was approved by the Institutional Animal Care and Use Committee (IACUC) of the Yangzhou University Animal Experiments Ethics Committee (permit number: SYXK (Su) IACUC2012-0029). Written informed consent was obtained from the owners for the participation of their animals in this study.

## Author Contributions

WB and SW conceived and supervised the study. YX and WB designed the experiments. YX and YZ performed the experiments. YX and SS analyzed the data. YX, HW, and WB contributed to the writing of the manuscript. All authors contributed to the article and approved the submitted version.

## Conflict of Interest

The authors declare that the research was conducted in the absence of any commercial or financial relationships that could be construed as a potential conflict of interest.
