# Supplementary material for: Effect of Promoter Methylation on the Expression of Porcine MUC2 Gene and Resistance to PEDV Infection
Source: Front Vet Sci. 2021 Apr 29;8:646408. doi: 10.3389/fvets.2021.646408 (PMC8116951; doi:10.3389/fvets.2021.646408)
Supplement: Supplementary file 1 [file Data_Sheet_1.PDF]

Effect analysis of promoter methylation on porcine *MUC2* gene expression and the resistance to PEDV infection

Yeyi Xiao[1†], Yajing Zhou[1†], Shouyong Sun[1], Haifei Wang[1], Shenglong Wu[1,2], Wenbin Bao[1,2*]

Supplementary materials

Table S1 Pathogen Primers information

Virus	Sequence of the primer	Length
PEDV-M	F: 5′-AGGTCTGCATTCCAGTGCTT-3′<br>R: 5′-GGACATAGAAAGCCCAACCA-3′	216 bp
TGEV-S1	F: 5'-CCAAACAGCCGTTATTAGTTA-3′<br>R: 5'-AGTGACACCACCCGTTGT-3'	218 bp
PoRV -VP6	F: 5'-CAAACGGGAGGAATAGGAA-3'<br>R: 5'-CACTCTTGGGAAACTGAACC-3'	572 bp
PDCoV-N	F: 5'-CCAAACGCAACCCCAACAATCC-3'<br>R: 5'- CTTCTCAGTGTCTGCAGAGCCG-3'	329 bp

Table S2 Real-time PCR primers

Gene	Accession number	Primer	Length
MUC2	NC_010444.4	F:5'-CAACCACCACTTCCACTC-3′<br>R:5′-AACTCACAGATGTCCTCAAG-3′	205 bp
GAPDH	AF017079.1	F:5′-ACATCATCCCTGCTTCTACTGG-3′<br>R:5′-CTCGGACGCCTGCTTCAC-3′	187 bp
ACTB	XM_00312428.3	F:5′-TGGCGCCCAGCACGATGAAG-3′<br>R:5′-GATGGAGGGGCCGGACTCGT-3′	149 bp

Table S3 Sequences of interfering RNA

siRNAs	Primer
si-*MUC2*-1	5′-CCGCCAAGCUCUGCCCUAATT-3′
si-*MUC2*-2	5′-GCGAGCAAUGUGUCUGCAATT-3′
si-*MUC2*-3	5′-CCACUCUACCAACCACCAUTT-3′
si-*MUC2*-NC	5′-UUCUCCGAACGUGUCACGUTT-3′
si-*YY1*-1	5′-CCGAGUACAUGACAGGAAATT-3′
si-*YY1*-2	5′-GCUCCAAGAACAAUAGCUUTT-3′
si-*YY1*-3	5′-GCAACUCAAGUUUCCUUUATT-3′
si-*YY1*-NC	5′-UUCUCCGAACGUGUCACGUTT-3′

Table S4 Primer information

Gene	Primer	Annealing temperature	Length
MUC2 （BSP）	F:5’-GTTTTGGGTTTTAGGGAGATATTTTAT-3’<br>R:5’-AACACTTACCAATTTATCAAAAAAACAA-3’	52	316
MUC2-1	F:5’-CGGGGTACCATGCGCTTCCGGCAGGAC-3’<br>R:5’-CTAGCTAGCCGGCAGCCCGATTACCCA-3’	60	122
MUC2 （ChIP-PCR）	F:5’-ATGCGCTTCCGGCAGGAC-3’<br>R:5’-CGGCAGCCCGATTACCCA-3’	60	104

MUC2-1 stands for the amplified primer for the mC-5 segment of the promoter region.

Table S5 List of Non-standard Abbreviations

Full Names	Abbreviations
Mucin 2	MUC2
Porcine epidemic diarrhea virus	PEDV
Transmissible gastroenteritis virus	TGEV
Porcine rotavirus	PoRV
Porcine deltacoronavirus	PDCoV
Porcine small intestinal epithelial cells	IPEC-J2
Transcription factor Yin Yang 1	YY1
Chromatin immunoprecipitation-PCR	CHIP-PCR

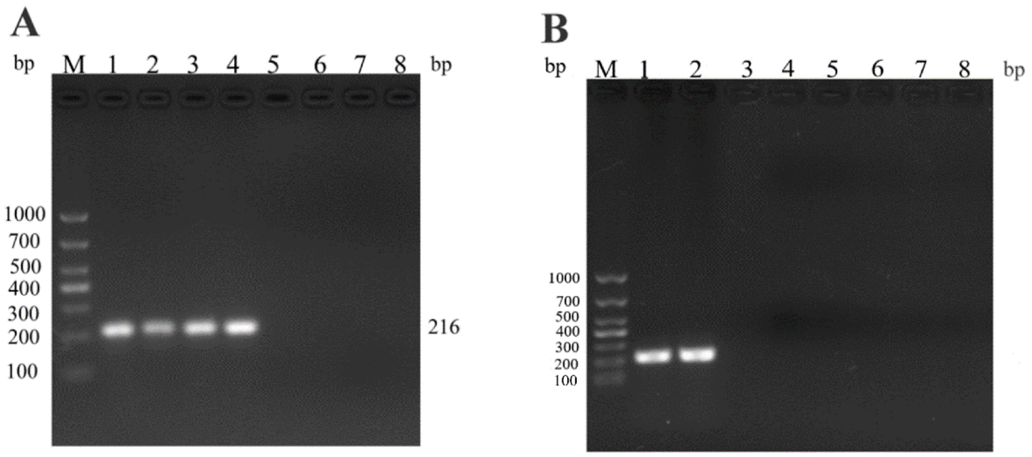

Fig. S1 Pathogen detection

A: PCR amplification results of *PEDV-M* gene, 1~4: porcine epidemic diarrhea individuals, 5~8: normal piglet individuals, M: DL1000 Marker; B: PCR amplification results of different virus, 1~2: *PEDV-M* gene, 3~4: *TGEV-S* gene, 5~6: *PoRV-VP6* gene, 7~8: *PDCoV-N* gene, M: DL 1000 Marker

A

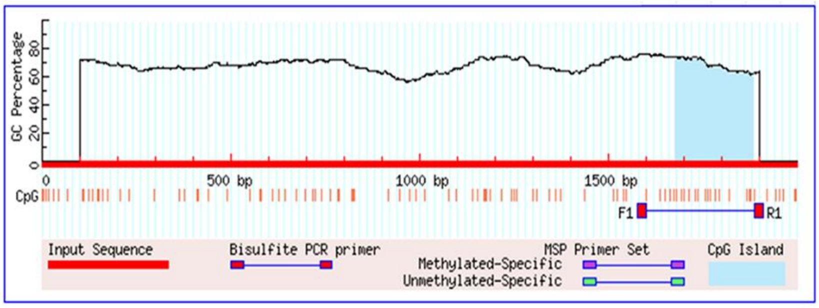

B

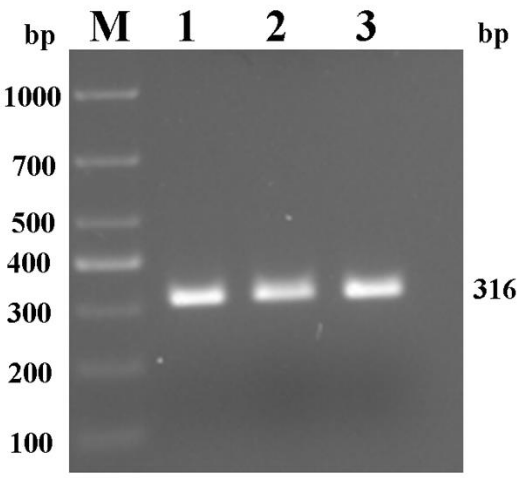

Fig. S2 Results of prediction of CpG islands in *MUC2* gene promoter regions and PCR amplification

A: CpG island in *MUC2* gene promoter region; B: PCR amplified fragment of CpG island in *MUC2* gene promoter region.

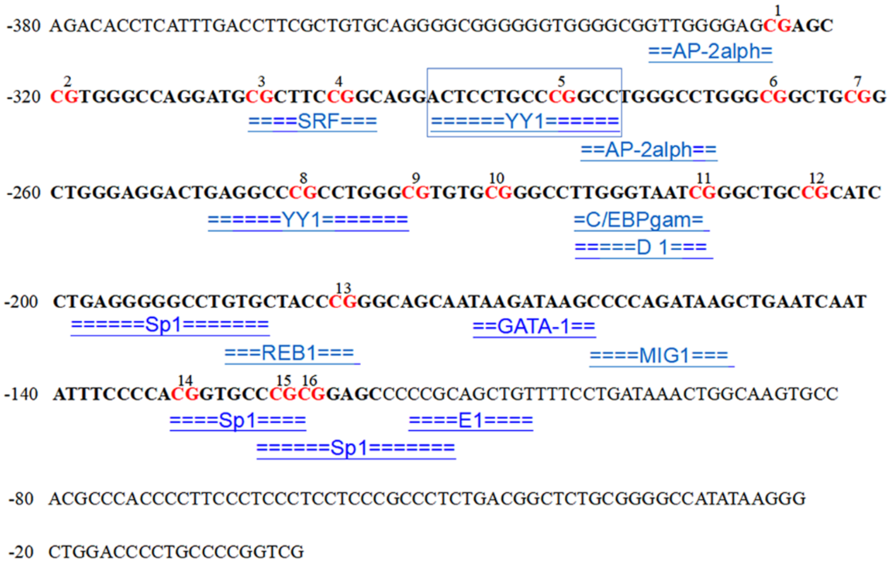

Fig. S3 Prediction of transcription factors in CpG island of *MUC2* promoter region
